# High SARS-CoV-2 Seroprevalence in Rural Peru, 2021: a Cross-Sectional Population-Based Study

**DOI:** 10.1128/mSphere.00685-21

**Published:** 2021-11-24

**Authors:** Andres Moreira-Soto, Johanna Maribel Pachamora Diaz, Lilian González-Auza, Xiomara Jeanleny Merino Merino, Alvaro Schwalb, Christian Drosten, Eduardo Gotuzzo, Michael Talledo, Heriberto Arévalo Ramirez, Roxana Peralta Delgado, Spassky Bocanegra Vargas, Jan Felix Drexler

**Affiliations:** a Charité-Universitätsmedizin Berlin, corporate member of Freie Universität Berlin and Humboldt-Universität zu Berlin, Institute of Virology, Berlin, Germany; b Virology-CIET, Faculty of Microbiology, University of Costa Rica, San José, Costa Rica; c Dirección Regional de Salud de San Martín, Moyobamba, Peru; d Instituto de Medicina Tropical Alexander von Humboldt, Universidad Peruana Cayetano Heredia, Lima, Peru; e Departamento de Medicina, Facultad de Medicina, Universidad Peruana Cayetano Heredia, Lima, Peru; f Moyobamba Hospital, Moyobamba, Peru; g German Centre for Infection Research (DZIF), associated partner site Charité, Berlin, Germany; University of Pittsburgh School of Medicine

**Keywords:** SARS-CoV-2, Peru, COVID-19, serology, seroprevalence, rural, Latin America

## Abstract

Latin America has been severely affected by the COVID-19 pandemic. The COVID-19 burden in rural settings in Latin America is unclear. We performed a cross-sectional, population-based, random-selection SARS-CoV-2 serologic study during March 2021 in the rural population of San Martin region, northern Peru. In total, 563 persons from 288 houses across 10 provinces were enrolled, reaching 0.2% of the total rural population of San Martin. Screening for SARS-CoV-2 IgG antibodies was done using a chemiluminescence immunoassay (CLIA), and reactive sera were confirmed using a SARS-CoV-2 surrogate virus neutralization test (sVNT). Validation of the testing algorithm using prepandemic sera from two regions of Peru showed false-positive results in the CLIA (23/84 sera; 27%) but not in the sVNT, highlighting the pitfalls of SARS-CoV-2 antibody testing in tropical regions and the high specificity of the two-step algorithm used in this study. An overall 59.0% seroprevalence (95% confidence interval [CI], 55 to 63%) corroborated intense SARS-CoV-2 spread in San Martin. Seroprevalence rates between the 10 provinces varied from 41.3 to 74.0% (95% CI, 30 to 84%). Higher seroprevalence was not associated with population size, population density, surface area, mean altitude, or poverty index in Spearman correlations. Seroprevalence and reported incidence diverged substantially between provinces, suggesting regional biases of COVID-19 surveillance data. Potentially, limited health care access due to environmental, economic, and cultural factors might lead to undetected infections in rural populations. Additionally, test avoidance to evade mandatory quarantine might affect rural regions more than urban regions. Serologic diagnostics should be pursued in resource-limited settings to inform country-level surveillance and vaccination strategies and to support control measures for COVID-19.

**IMPORTANCE** Latin America is a global hot spot of the COVID-19 pandemic. Serologic studies in Latin America have been mostly performed in urban settings. Rural populations comprise 20% of the total Latin American population. Nevertheless, information on COVID-19 spread in rural settings is scarce. Using a representative population-based seroprevalence study, we detected a high seroprevalence in rural populations in San Martin, northern Peru, in 2021, reaching 41 to 74%. However, seroprevalence and reported incidence diverged substantially between regions, potentially due to limited health care access or test avoidance due to mandatory quarantine. Our results suggest that rural populations are highly affected by SARS-CoV-2 even though they are sociodemographically distinct from urban populations and that highly specific serological diagnostics should be performed in resource-limited settings to support public health strategies of COVID-19 control.

## OBSERVATION

Peru has been severely affected by the COVID-19 pandemic, with one of the highest mortality per capita reported worldwide since the start of the pandemic, reaching 6,132 deaths per million as of October 2021 (1, 2). The determinants of SARS-CoV-2 spread in Latin America are poorly defined. An epidemiological study from European and Asian urban centers correlated population density with increased SARS-CoV-2 infection rates (3). In contrast, epidemiological studies from Latin American urban centers yielded diverse prevalence estimates that seemed uncorrelated with population density, exemplified by the three available seroprevalence studies from Peru, conducted during mid- to late 2020. The first study from the Peruvian capital, Lima (population, 9.5 million; density, 12,000/km^2^), reported a 21% seroprevalence rate (4). The second study from the Lambayeque department in northern Peru (population, 1.2 million; density, 84/km^2^) reported a 29% seroprevalence rate (5). The third study from Iquitos city, Peruvian Amazon (population, 470,000; density, 417/km^2^), reported a 70% seroprevalence rate, implying local herd immunity had been reached (6). Despite the high seroprevalence already during 2020, a second SARS-CoV-2 wave occurred in Iquitos in January 2021. Potentially, mobile susceptible populations in the Amazon region might explain the occurrence of a second wave in Iquitos (6). Susceptible populations might correspond to rural and indigenous populations, comprising up to 19% of the total Latin American population based on World Bank estimates (https://data.worldbank.org/indicator/SP.RUR.TOTL.ZS?locations=ZJ). The high geographical dispersion and low population density of these communities, in addition to regular traveling to urban centers for commerce, might contribute to a differential dispersion of SARS-CoV-2 compared to urban settings.

### Seroprevalence study in San Martin.

**FIG 1:**
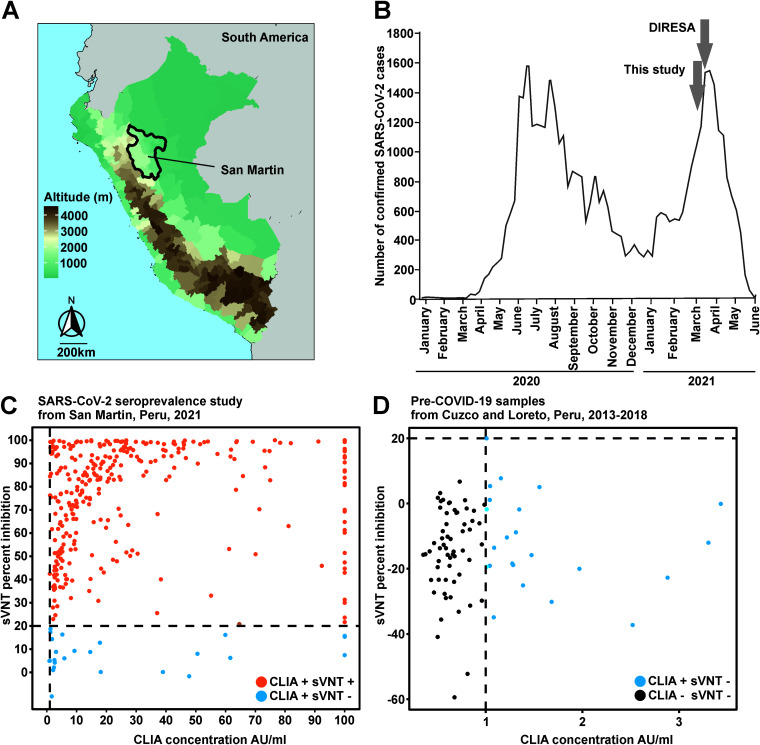
Epidemiological surveillance and serologic diagnostics of SARS-CoV-2 from San Martin, Peru. (A) Mean altitude (meters above sea level) map of Peru. The department of San Martin is circled in black. (B) Number of confirmed cases in San Martin as of June 2021. The times where incidence data and samples for the serologic study were taken are marked with arrows. Surveillance data were gathered from https://diressanmartin.gob.pe/ and https://www.gob.pe/minsa/. Reactivity of serum samples from SARS-CoV-2 seroprevalence study in San Martin in 2021 (C) and pre-COVID-19 samples from 2013 to 2018 from Peru (D) in a chemiluminescence immunoassay (CLIA), shown in the *x* axis, and a SARS-CoV-2 surrogate virus neutralization test (sVNT), shown in the *y* axis. AU/ml, absorbance unit per milliliter.

Our study focused on San Martin, Peru (total population, 899,648; density, 18/km^2^). The San Martin department (51,000 km^2^) is located in the north of Peru (Fig. 1A). The department encompasses diverse ecosystems such as valleys, Andes mountains, and Amazon rainforests, reaching altitudes from 190 to 3,080 m (Fig. 1A), and comprises ≤5% of Peruvian forest cover. The San Martin population is divided into Mestizo (83.6%) followed by Quechua (5.3%) and Afro-Peruvian (4.9%) ethnicities per census data of the National Institute of Statistics and Census of Peru (INEI; www.inei.gob.pe/).

We performed a cross-sectional population-based random-selection study during March 2021 (institutional ethics committee Via Libre; number 6532-2021a) in the 10 provinces of San Martin (Fig. 1B). Sample size calculation considered the rural population of San Martin (286,988), a 95% confidence level, and an estimated SARS-CoV-2 seroprevalence of 50%, reaching 576 individuals. As the average household in Peru consists of 4 inhabitants (www.inei.gob.pe/), the study aimed at sampling two individuals from 288 individual houses. First, a conglomerate of 100 rural houses was selected based on estimates of the census data of 2017 and on geographic information systems of the INEI. Next, a random household selection in the conglomerate was performed. Exclusion criteria encompassed populations residing in collective dwellings such as barracks, police stations, convents, boarding schools, and hotels, used as temporary housing of unrelated people with unstable population dynamics; age ≤5 years; skin lesions in the venous puncture site; use of alcohol or psychoactive drugs; not being a permanent resident; and not signing the informed consent form. Participation was voluntary, and participant selection in households was performed using a Kish grid (7). A 10-ml blood sample was taken after the informed consent was signed by the person or the caretaker if the person was ≤18 years of age. We obtained samples from 563 persons visiting 288 houses in the 10 provinces, comprising 0.2% of the total rural population of San Martin. The sampled cohort included persons aged 6 to 89 years (mean, 35.8; standard deviation [SD]: 21.15) consistent with San Martin’s age distribution data from the INEI 2017 census (see Fig. S1 in the supplemental material; www.inei.gob.pe/). From the cohort, 37.7% (212/563) were male and 62.3% (351/563) female, contrasting with the sex distribution in San Martin (51.3% male, 48.7% female). The difference between the sex distribution can be explained by the sampling strategy, limited to visiting the homes of the participants during the day due to safety and operational reasons, while males were mostly out working.

10.1128/mSphere.00685-21.1 FIG S1 Age distribution from census data in San Martin in comparison to this study. Data from the National Institute of Statistics and Informatics of Peru (https://www.inei.gob.pe/). Download FIG S1, TIF file, 1.8 MB. Copyright © 2021 Moreira-Soto et al. 2021 Moreira-Soto et al. https://creativecommons.org/licenses/by/4.0/ This content is distributed under the terms of the Creative Commons Attribution 4.0 International license.

### Two-step serologic testing and validation.

Using a chemiluminescence immunoassay (CLIA) (SARS-CoV-2 S-RBD IgG kit; Snibe Diagnostic, China), we detected SARS-CoV-2-specific IgG antibodies in 63.6% of the samples (358/563) (Fig. 1C and Fig. S2). To validate the CLIA results, we used 84 pre-COVID-19 samples from our previous study of arbovirus serology in Peru (8) (Fig. 1D and Fig. S2). The samples were collected in the departments of Cuzco and Loreto during 2013 and 2018, respectively. A total of 27% (23/84; 11 from Cuzco and 12 from Loreto) of pre-COVID-19 samples yielded positive results in the CLIA irrespective of geographical location (Fig. 1D and Fig. S2). The apparently positive pre-COVID-19 samples showed statistically significantly lower signal concentrations in the CLIA than those samples from 2021 (median pre-COVID-19 = 1.6 versus COVID-19 = 32.81; *t* test, *P* < 0.001) (Fig. S2), suggesting unspecific reactivity. Unspecific reactivity may be elicited by antibodies against common-cold coronaviruses (9) or endemic tropical diseases such as malaria, dengue, and Zika, either by polylconal B-cell stimulation or by weakly cross-reactive antibodies (10, 11). Therefore, a confirmatory SARS-CoV-2 surrogate virus neutralization test (sVNT; GenScript, USA) was performed in all CLIA-reactive samples. A total of 92.7% (332/358) of the 2021 CLIA-positive samples were confirmed using the sVNT, whereby minor differences between CLIA and sVNT results can be attributed to differential sensitivity of the tests. (Fig. 1C and Fig. S2). In stark contrast, none of the CLIA-positive prepandemic samples yielded positive results in the sVNT. These results corroborate that unspecific reactivity of serologic tests in tropical areas must be carefully evaluated (10) and highlight the robustness of our serologic testing algorithm. Therefore, only samples yielding positive results in both serologic assays were considered for further analyses in our study to maximize specificity.

10.1128/mSphere.00685-21.2 FIG S2 Validation of the testing algorithm using pre-COVID-19 samples from Peru. CLIA concentration comparison between SARS-CoV-2-positive samples from the 2021 cohort from this study and 84 pre-COVID-19 samples from Peru in 2013 and 2018. Line inside the box plots denotes the median. N.s., not significant. Download FIG S2, TIF file, 1.8 MB. Copyright © 2021 Moreira-Soto et al. 2021 Moreira-Soto et al. https://creativecommons.org/licenses/by/4.0/ This content is distributed under the terms of the Creative Commons Attribution 4.0 International license.

### Seroprevalence and statistical analyses.

Overall, the SARS-CoV-2 seroprevalence for San Martin was 59.0% (332/563; 95% confidence interval [CI], 55 to 63%). No statistically significant difference of seroprevalence per sex was observed, using a chi-square test (χ^2^ = 0.05; *P* = 0.83). However, a statistically significant difference was observed between the ages of SARS-CoV-2-seropositive and SARS-CoV-2-seronegative persons using a *t* test (33 years [range, 6 to 89] versus 38 years [range, 7 to 57; *P* = 0.01]). This observation was in concordance with the previously mentioned study from Iquitos, likely associated with higher contact rates facilitating SARS-CoV-2 transmission in younger age groups (6).

**FIG 2:**
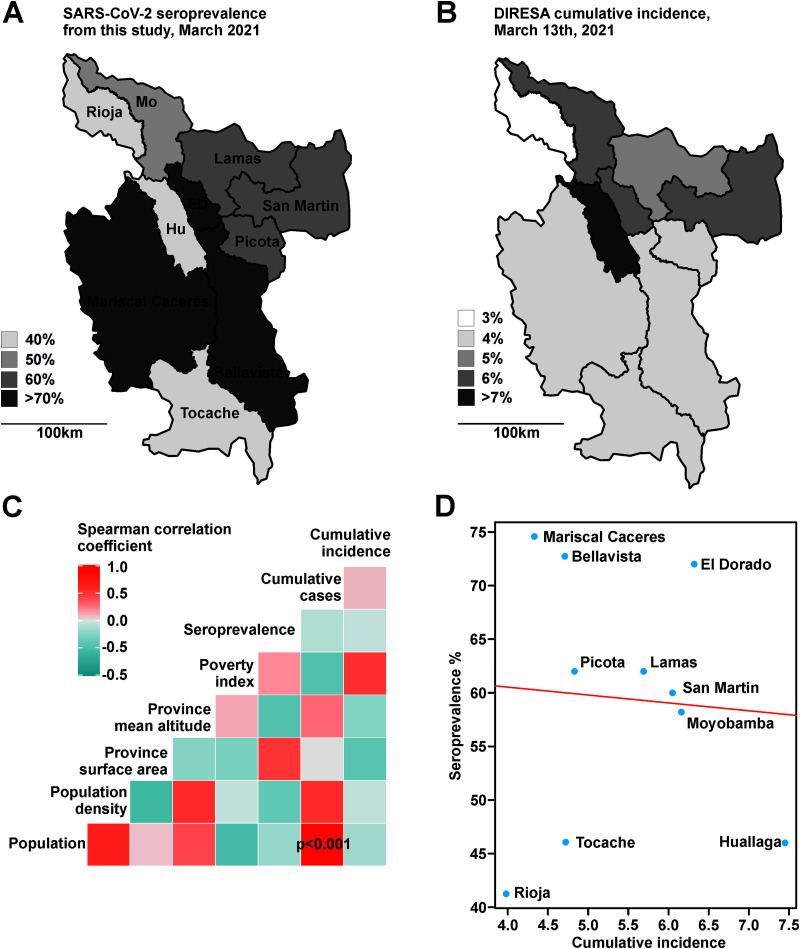
Correlation of seroprevalence and incidence data with different indicators. (A and B) Comparison of serologic (A) and incidence (B) data from San Martin. Hu, Huallaga; Mo, Moyobamba; ED, El Dorado. (C) Heatmap of Spearman’s rank correlation test using different social, economic, and geographical indicators. Significant correlations are depicted inside the square. (D) Spearman’s rank correlation test of seroprevalence and cumulative incidence per province.

Seroprevalence between the 10 provinces varied from 41.3 to 74.0% (CI, 30 to 84%) (Fig. 2A and B and Table S1). Higher seroprevalence was neither associated with population size (Spearman correlation test, *r_s_* = −0.26; *P* = 0.46), population density (*r_s_* = −0.14; *P* = 0.68), surface area (*r_s_* = 0.33; *P* = 0.34), mean altitude of the province (*r_s_* = −0.59; *P* = 0.06), nor poverty index (*r_s_* = 0.10; *P* = 0.76) (Fig. 2C and Fig. S3). Interestingly, the cumulative epidemiological surveillance data from the Regional Health Directorate of Peru (DIRESA) relying on notified cases of acute SARS-CoV-2 infection confirmed by either reverse transcription-PCR (RT-PCR) or an antigen test (Fig. 2B) showed no statistically significant correlation with our serologic data although cumulatively measuring similar time spans, encompassing early 2020 to March 2021 (*r_s_* = −0.14; *P* = 0.68) (Fig. 1B and Fig. 2D). On the one hand, overall higher seroprevalence than reported cases is consistent with high numbers of undetected asymptomatic COVID-19 cases (12). On the other hand, we detected two provinces with a relatively higher divergence of seroprevalence compared to incidence data (Mariscal Caceres and Bellavista, Fig. 2A, B, and D). Hypothetically, individuals in those provinces may lack health care access due to numerous environmental, economic, and cultural factors (13), which might lead to more undetected infections in these rural populations. First, test refusal to avoid mandatory quarantine or stigmatization has been reported for SARS-CoV-2 from different countries (14, 15). Test avoidance might be greater in rural regions of resource-limited areas, where a high share of the population work under conditions that are not compatible with quarantine and hospital visits. Second, local health centers might be difficult to access or not be sufficiently equipped for the high COVID-19 burden (16), limiting access to diagnostics in rural areas. Third, high infection rates in rural populations might be explained by lack of access to basic housing elements such as sewage disposal systems, as observed in rural populations in Ecuador (17). Similarly, a survey analyzing rural residents in the United States found that they were less likely to participate in COVID-19 preventive measures such as the use of masks in public or working from home, increasing the possibility of infection (18). Another province (Huallaga, Fig. 2A, B, and D) showed higher cumulative incidence than other provinces for unknown reasons, substantiating inconsistencies between serologic and incidence data.

10.1128/mSphere.00685-21.3 FIG S3 Spearman’s rank correlation test of seroprevalence and population, population density, surface area, poverty index, and altitude. Rs, Spearman’s correlation coefficient. Epidemiological data taken from http://sial.minam.gob.pe/sanmartin/indicador/850 and https://www.citypopulation.de/en/peru/sanmartin/admin/. Download FIG S3, TIF file, 1.9 MB. Copyright © 2021 Moreira-Soto et al. 2021 Moreira-Soto et al. https://creativecommons.org/licenses/by/4.0/ This content is distributed under the terms of the Creative Commons Attribution 4.0 International license.

10.1128/mSphere.00685-21.4 TABLE S1 Demographics per province, seropositivity, and incidence data from San Martin, Peru, 2021. Download Table S1, DOCX file, 0.02 MB. Copyright © 2021 Moreira-Soto et al. 2021 Moreira-Soto et al. https://creativecommons.org/licenses/by/4.0/ This content is distributed under the terms of the Creative Commons Attribution 4.0 International license.

Irrespective of the underlying reasons leading to the overall high seroprevalence, our data suggest a large number of undiagnosed COVID-19 cases potentially challenging test-trace-isolate interventions in the region (19). Previous studies in the United States have stressed that rural regions are particularly vulnerable to COVID-19, leading to higher mortality rates in rural than in urban regions, and with higher mortality rates associated with black and Hispanic populations (20, 21), suggesting further studies are needed in Latin American vulnerable populations. Limitations of our study include limited metadata and potential sampling biases. However, thorough study design, exhaustive serologic testing, and high seroprevalence consistent with data from other Latin American settings suggest robustness of our results (6, 17). Serologic diagnostics should be pursued in resource-limited settings to inform country-level surveillance and vaccination strategies and to support control measures for COVID-19.
