# Supplementary figures and images for: High SARS-CoV-2 Seroprevalence in Rural Peru, 2021: a Cross-Sectional Population-Based Study
Source: mSphere. 2021 Nov 24;6(6):e00685-21. doi: 10.1128/mSphere.00685-21 (PMC8612248; doi:10.1128/mSphere.00685-21)

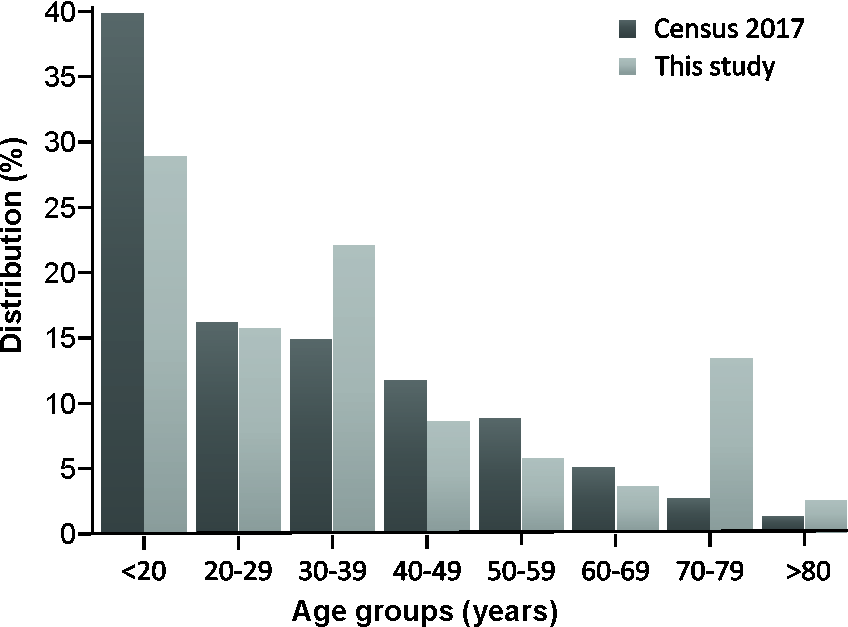

Supplement: FIG S1 [file msphere.00685-21-sf001.tif]

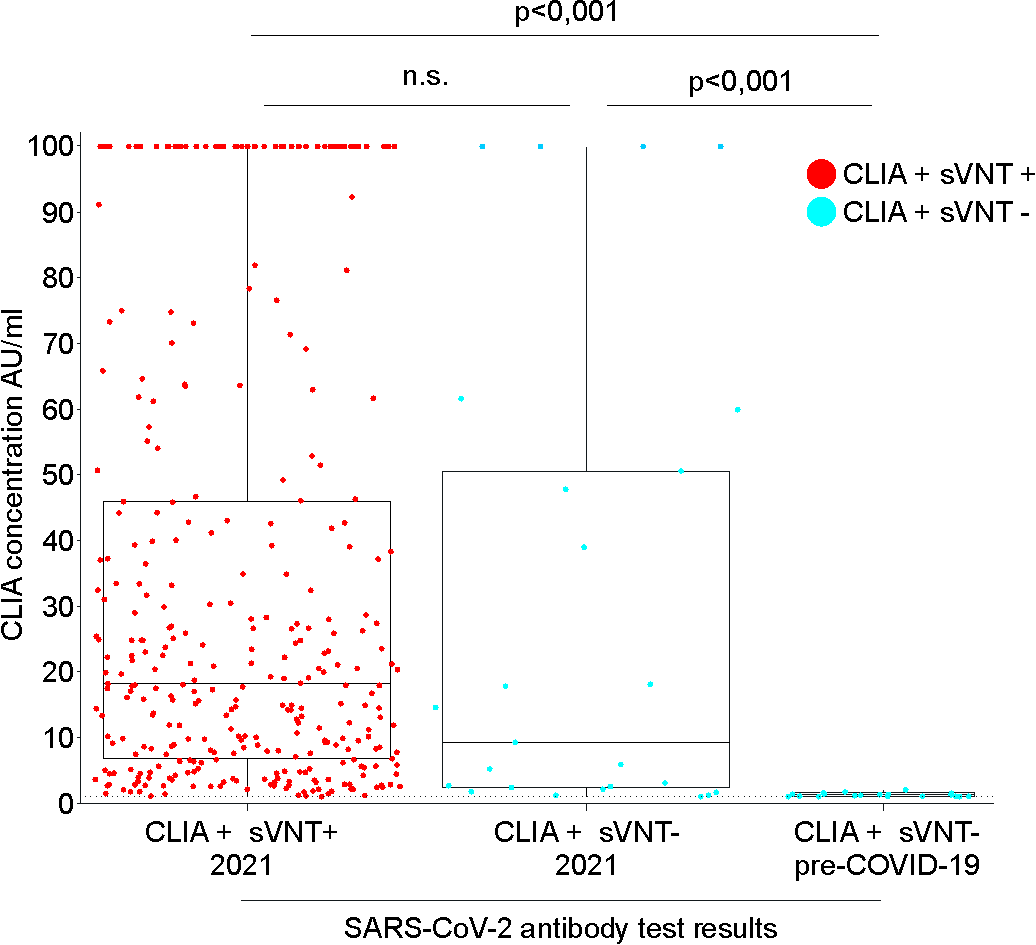

Supplement: FIG S2 [file msphere.00685-21-sf002.tif]

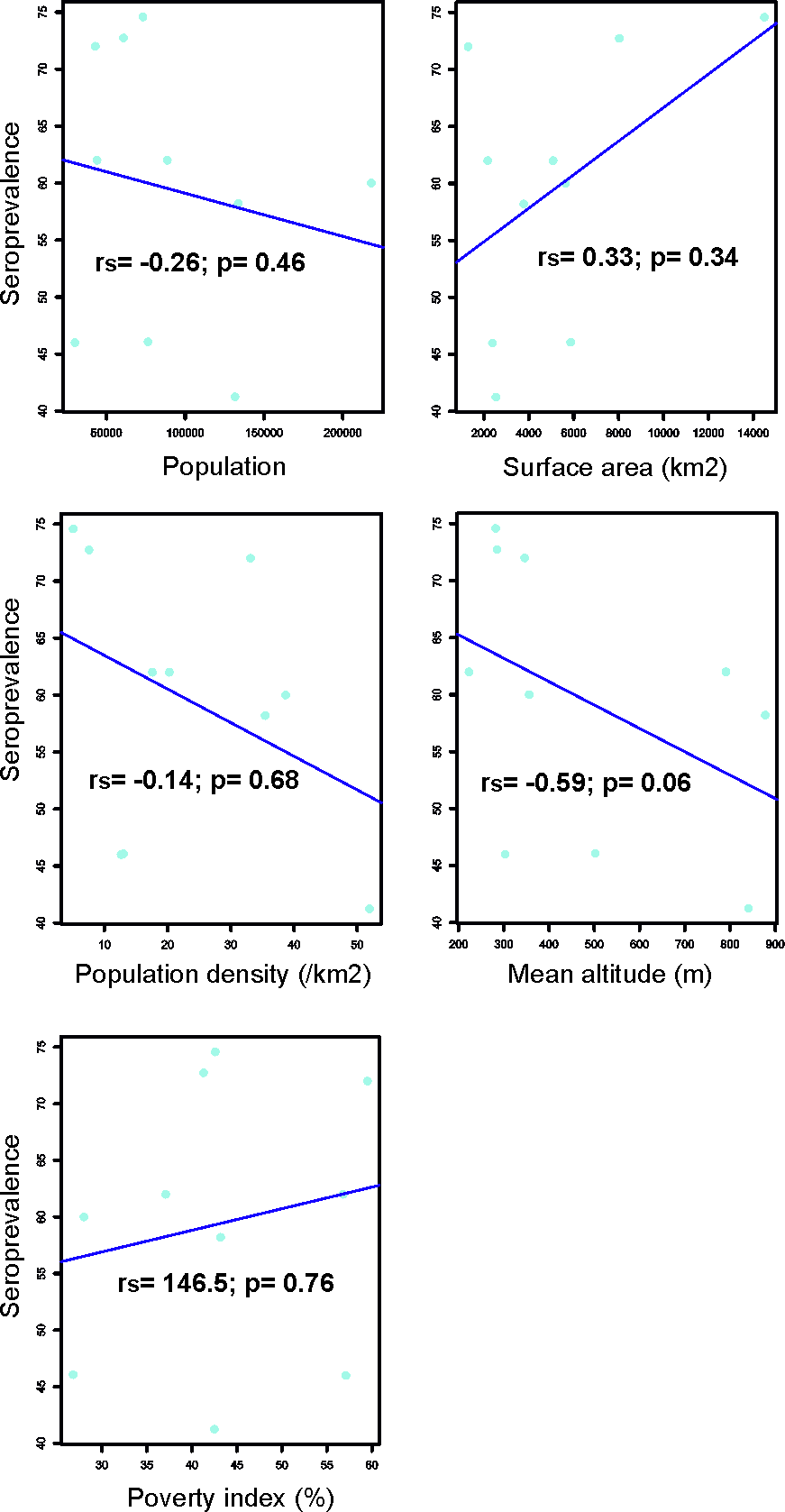

Supplement: FIG S3 [file msphere.00685-21-sf003.tif]
