# Supplementary material for: High SARS-CoV-2 Seroprevalence in Rural Peru, 2021: a Cross-Sectional Population-Based Study
Source: mSphere. 2021 Nov 24;6(6):e00685-21. doi: 10.1128/mSphere.00685-21 (PMC8612248; doi:10.1128/mSphere.00685-21)
Supplement: TABLE S1 [file msphere.00685-21-st001.docx]

**Annex Table 1.** Demographics per province, seropositivity and incidence data from San Martin, Peru, 2021

| **Province** | **Population (Projection 2020)*** | **Population density (/ km^2)^*** | **Surface area ( km2)** | **Mean altitude (m)** | **Poverty index (%)^$^** | **N° positives / Total analyzed** | **Seroprevalence % (Confidence Interval)** | **DIRESA** cumulative cases** | **DIRESA cumulative incidence (%) ^#^** |
| --- | --- | --- | --- | --- | --- | --- | --- | --- | --- |
| **Rioja** | 131 651 | 51.93 | 2 535.04 | 841 | 42.50 | 33/80 | 41.25  (31-52) | 5 240 | 3.98 |
| **Huallaga** | 30 097 | 12.64 | 2 380.85 | 303 | 57.10 | 23/50 | 46.00  (33-59) | 2 242 | 7.45 |
| **Tocache** | 76 450 | 13.03 | 5 865.44 | 502 | 26.80 | 14/30 | 46.70  (30-63) | 3 610 | 4.72 |
| **Moyobamba** | 133 631 | 35.42 | 3 772.31 | 878 | 43.20 | 46/79 | 58.20  (47-68) | 6 903 | 5.16 |
| **San Martin** | 218 074 | 38.67 | 5 639.82 | 356 | 28.00 | 30/50 | 60.00  (46-72) | 13 188 | 6.05 |
| **Picota** | 44 039 | 20.28 | 2 171.41 | 223 | 37.10 | 31/50 | 62.00  (48-74) | 2 128 | 4.83 |
| **Lamas** | 88 637 | 17.58 | 5 082.54 | 791 | 56.80 | 50/80 | 62.00  (52-72) | 5 050 | 5,69 |
| **El Dorado** | 42 983 | 33.11 | 1 298.14 | 346 | 59.50 | 36/50 | 72.00  (58-83) | 2 719 | 6.32 |
| **Bellavista** | 60 893 | 7.56 | 8 033.19 | 285 | 41.30 | 32/44 | 72.73  (58-83) | 2 868 | 4.71 |
| **Mariscal Caceres** | 73 193 | 5.05 | 14 498.73 | 282 | 42.60 | 37/50 | 74.00  (60-84) | 3 168 | 4.33 |
| **Total** | 899 648 | 17.55 | 51 253.31 | 1609 | 43.49 | 332/563 | 58.97  (55-63) | 47 116 | 12.67 |

*2020 projection data gathered from the Peruvian National Institute of Statistics and Informatics (inei.gob.pe). ** DIRESA: Regional Health Directorate of Peru. #Cumulative incidence was calculated with the cumulative cases/ total population per province. $Poverty index is calculated as the percent of the population that have salaries below the regional poverty line corresponding to 328 Soles/monthly/per capita, corresponding to the minimum wage for their nutritional and non-nutritional necessities. Taken from INEI.gob.pe and from the Regional government of San Martin.
